# Mechanism of photocatalytic water oxidation on small TiO_2_ nanoparticles†
†Electronic supplementary information (ESI) available: Full details on computational methodology, analysis of reactive trajectory for (TiO_2_)_4_(OH)_4_(H_2_O)_4_ and of non-adiabatic transitions. See DOI: 10.1039/c6sc04378j



**DOI:** 10.1039/c6sc04378j

**Published:** 2016-12-07

**Authors:** Mikko Muuronen, Shane M. Parker, Enrico Berardo, Alexander Le, Martijn A. Zwijnenburg, Filipp Furche

**Affiliations:** a Department of Chemistry , University of California , 1102 Natural Sciences II , Irvine , CA 92697-2025 , USA . Email: mmuurone@uci.edu ; Email: filipp.furche@uci.edu ; Fax: +1 949 824 8571 ; Tel: +1 949 824-5051; b Department of Chemistry , Imperial College London , South Kensington , London , SW7 2AZ , UK; c Department of Chemistry , University College London , 20 Gordon Street , London WC1H 0AJ , UK

## Abstract

We present the first unconstrained nonadiabatic molecular dynamics (NAMD) simulations of photocatalytic water oxidation by small hydrated TiO_2_ nanoparticles using Tully surface hopping and time-dependent density functional theory. The results indicate that ultrafast electron–proton transfer from physisorbed water to the photohole initiates the photo-oxidation on the S_1_ potential energy surface. The new mechanism readily explains the observation of mobile hydroxyl radicals in recent experiments. Two key driving forces for the photo-oxidation reaction are identified: localization of the electron–hole pair and stabilization of the photohole by hydrogen bonding interaction. Our findings illustrate the scope of recent advances in NAMD methods and emphasize the importance of explicit simulation of electronic excitations.

## Abstract 2

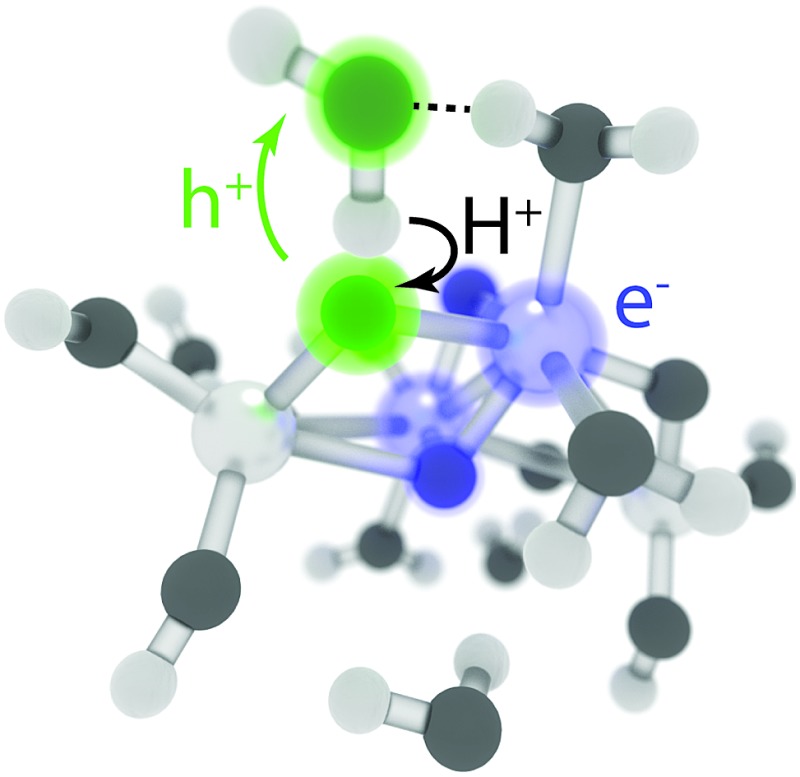
Nonadiabatic molecular dynamics simulations suggest an excited state electron proton transfer mechanism and explain the observation of mobile hydroxyl radicals.

## Introduction

TiO_2_ is the prototypical redox photocatalyst because it is inexpensive, abundant, versatile, and non-toxic.^1,2^ Ever since the ability of TiO_2_ to split water was discovered in 1972,^3^ the use of TiO_2_ for solar fuel generation has been intensely studied. However, the efficiency of TiO_2_-based photocatalysts has remained moderate, which was attributed to a lack of mechanistic understanding and models to inform synthetic improvements.^4^ Apart from sample preparation, heterogeneity, and system size, a key challenge of mechanistic studies of TiO_2_ photocatalysis are ultra-fast processes involving nonadiabatic transitions between electronic states, which are exceedingly difficult to characterize experimentally and theoretically.

Here we propose a detailed mechanistic model for photocatalytic water oxidation on TiO_2_ nanoparticles. Our model explicitly accounts for exciton dynamics, nonadiabatic transitions, and bond breaking for the first time, and is based on recent methodological developments of *on-the-fly* non-adiabatic molecular dynamics (NAMD) simulations.^5–8^

The high kinetic barrier for photolytic water splitting on TiO_2_ surfaces is caused by the oxygen evolution reaction (OER),^9–12^

1 2H_2_O + 4h^+^ → O_2_ + 4H^+^.

The first of the four one-electron oxidation steps is likely rate-limiting;^10,12–14^ however, the mechanism of this step is controversial and several conflicting models have been proposed. The earliest models based on spectroscopic experiments suggested that the photohole (h^+^) oxidizes a surface bound hydroxyl group <svg xmlns="http://www.w3.org/2000/svg" version="1.0" width="16.000000pt" height="16.000000pt" viewBox="0 0 16.000000 16.000000" preserveAspectRatio="xMidYMid meet"><metadata> Created by potrace 1.16, written by Peter Selinger 2001-2019 </metadata><g transform="translate(1.000000,15.000000) scale(0.005147,-0.005147)" fill="currentColor" stroke="none"><path d="M0 2080 l0 -80 80 0 80 0 0 -40 0 -40 160 0 160 0 0 -40 0 -40 160 0 160 0 0 -40 0 -40 160 0 160 0 0 -40 0 -40 160 0 160 0 0 -40 0 -40 160 0 160 0 0 -40 0 -40 160 0 160 0 0 -40 0 -40 160 0 160 0 0 -40 0 -40 160 0 160 0 0 80 0 80 -160 0 -160 0 0 40 0 40 -160 0 -160 0 0 40 0 40 -160 0 -160 0 0 40 0 40 -160 0 -160 0 0 40 0 40 -160 0 -160 0 0 40 0 40 -160 0 -160 0 0 40 0 40 -160 0 -160 0 0 40 0 40 -160 0 -160 0 0 40 0 40 -80 0 -80 0 0 -80z M2400 1000 l0 -40 -160 0 -160 0 0 -40 0 -40 -160 0 -160 0 0 -40 0 -40 -160 0 -160 0 0 -40 0 -40 -160 0 -160 0 0 -40 0 -40 -160 0 -160 0 0 -40 0 -40 -160 0 -160 0 0 -40 0 -40 -160 0 -160 0 0 -40 0 -40 -80 0 -80 0 0 -80 0 -80 80 0 80 0 0 40 0 40 160 0 160 0 0 40 0 40 160 0 160 0 0 40 0 40 160 0 160 0 0 40 0 40 160 0 160 0 0 40 0 40 160 0 160 0 0 40 0 40 160 0 160 0 0 40 0 40 160 0 160 0 0 40 0 40 160 0 160 0 0 80 0 80 -160 0 -160 0 0 -40z"/></g></svg> Ti^IV^–OH,^15,16^

2 Ti^IV^–OH + h^+^ → Ti^IV^–(·OH)^+^.

This mechanism was challenged by the results of density functional theory (DFT) calculations suggesting that Ti^IV^–OH groups trap electrons, not holes.^17^ An alternative mechanism,

3 Ti^IV^–OH_2_ + h^+^ → Ti^IV^–(·OH) + H^+^,

involves oxidation of a surface bound water Ti^IV^–OH_2_ instead of Ti^IV^–OH by the photohole.^10^ The high barrier of this step can be lowered by deprotonation of Ti^IV^–OH_2_ with base, resulting a barrierless hole transfer.^12,18,19^ The resulting proton-coupled electron transfer (PCET) mechanism, where a strongly localized photohole h^+^ is transferred from a bridging oxygen O_br_ to the Ti^IV^–(OH)^–^ species, is consistent with the observed pH dependence of the OER.^9,20^ However, the recent experimental detection of mobile rather than surface bound OH radicals in three different experiments casts doubts on the hypothesis that the oxidized water is bound to the TiO_2_ surface.^21–24^ Based on scanning tunneling microscopy (STM) experiments, Hou and co-workers suggested that mobile ·OH species result from protonated O_br_ sites as a reaction intermediate:^22^

4 Ti^IV^–OH_2_ + h^+^ → Ti^IV^–(O_br_H)^+^–Ti^IV^


<svg xmlns="http://www.w3.org/2000/svg" version="1.0" width="16.000000pt" height="16.000000pt" viewBox="0 0 16.000000 16.000000" preserveAspectRatio="xMidYMid meet"><metadata>
Created by potrace 1.16, written by Peter Selinger 2001-2019
</metadata><g transform="translate(1.000000,15.000000) scale(0.005147,-0.005147)" fill="currentColor" stroke="none"><path d="M2560 2120 l0 -40 -160 0 -160 0 0 -40 0 -40 -160 0 -160 0 0 -40 0 -40 -160 0 -160 0 0 -40 0 -40 -160 0 -160 0 0 -40 0 -40 -160 0 -160 0 0 -40 0 -40 -160 0 -160 0 0 -40 0 -40 -160 0 -160 0 0 -40 0 -40 -160 0 -160 0 0 -80 0 -80 160 0 160 0 0 40 0 40 160 0 160 0 0 40 0 40 160 0 160 0 0 40 0 40 160 0 160 0 0 40 0 40 160 0 160 0 0 40 0 40 160 0 160 0 0 40 0 40 160 0 160 0 0 40 0 40 160 0 160 0 0 40 0 40 80 0 80 0 0 80 0 80 -80 0 -80 0 0 -40z M0 960 l0 -80 160 0 160 0 0 -40 0 -40 160 0 160 0 0 -40 0 -40 160 0 160 0 0 -40 0 -40 160 0 160 0 0 -40 0 -40 160 0 160 0 0 -40 0 -40 160 0 160 0 0 -40 0 -40 160 0 160 0 0 -40 0 -40 160 0 160 0 0 -40 0 -40 80 0 80 0 0 80 0 80 -80 0 -80 0 0 40 0 40 -160 0 -160 0 0 40 0 40 -160 0 -160 0 0 40 0 40 -160 0 -160 0 0 40 0 40 -160 0 -160 0 0 40 0 40 -160 0 -160 0 0 40 0 40 -160 0 -160 0 0 40 0 40 -160 0 -160 0 0 40 0 40 -160 0 -160 0 0 -80z"/></g></svg>

 + ·OH.

Recent Ehrenfest NAMD simulations of periodic TiO_2_ surfaces also considered this mechanism,^25^ but the simulation times were too short (up to 20 fs) to be conclusive. Nakato and coworkers suggested that nucleophilic attack of water on a O_br_, activated by h^+^, might initiate the OER,^26,27^

5 O_br_(h^+^) + H_2_O(l) → ·O_br_OH + H^+^,

generating a surface-bound hydroperoxyl radical. Later, Imanishi and coworkers suggested that this mechanism will dominate at low and intermediate pH, while at high pH the photohole could readily oxidize the Ti–O^–^ species present in high pH.^20^ Based on a transition state (TS) study for Ti(OH)_4_, Kazaryan and coworkers questioned mechanism (5) and proposed that Ti(OH)_4_, the smallest model of a hydroxylated TiO_2_ surface, can readily oxidize H_2_O(l) in the S_1_ excited state *via* hydrogen transfer mechanism from H_2_O(l) to electronically excited Ti(OH)_4_,^28^

6

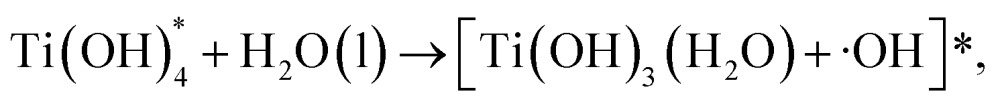

thus producing an intermediate similar to the one proposed in mechanism (4).

## Methods

The PBE0 (ref. 29) hybrid functional and polarized double-*ζ* valence def2-SVP^30^ basis sets were used for NAMD simulations. To account for van der Waals interactions, D3 dispersion corrections were employed.^31^ The forces on the S_1_ and S_0_ potential energy surfaces (PESs) and the non-adiabatic couplings between them were computed analytically at each time step.^32,33^ The nuclear dynamics used Tully's surface hopping algorithm and a leapfrog–Verlet integrator with time-step of 40 a.u. (1 fs).^34,35^ 115 trajectories were initiated with random nuclear velocities consistent with a 350 K thermal ensemble, and the trajectories were propagated for up to 1 ps. To describe homolytic bond cleavage, the spin symmetry was allowed to break if triplet instability was found for the reference state. We recently showed that this methodology can treat both closed and open shell pathways semiquantitatively in photodissociation of acetaldehyde.^8^ To avoid convergence and stability problems close to the conical intersections (CIs), a surface hop was forced if the S_1_–S_0_ gap is below 0.5 eV.^6,36^ All computations were performed using a local development version based on Turbomole 7.1.^37^ See ESI† for benchmark results and further details.

## Results and discussion

**Fig. 1:**
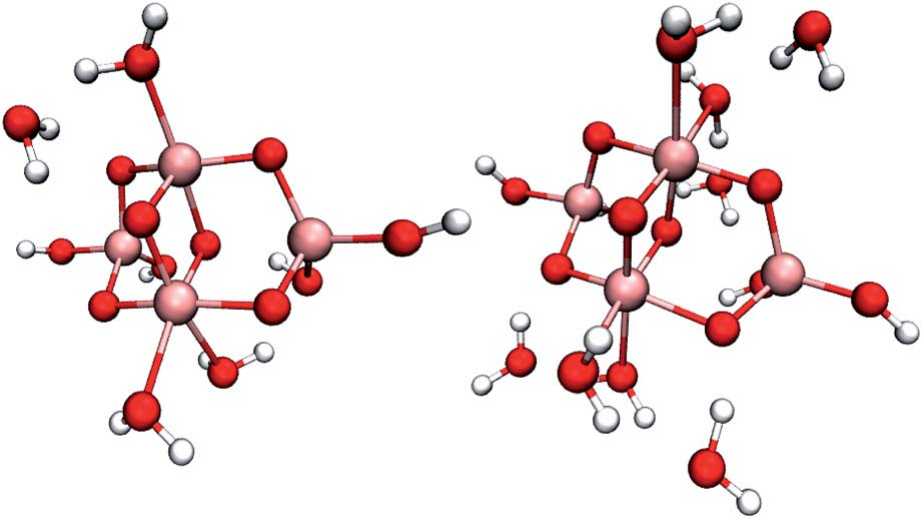
Studied (TiO_2_)_4_(OH)_4_ nanoparticles with four (left) and eight (right) additional water molecules. Pink, red and white spheres represent titanium, oxygen and hydrogen atoms, respectively.

To compare the reactivity of surface-bound *vs.* physisorbed water, we simulated small hydrated (TiO_2_)_4_(OH)_4_ nanoparticles with two, four, eight or ten additional water molecules, see Fig. 1 and S3.† These models can accommodate all proposed mechanisms (2)–(6) with the exception of mechanisms requiring deprotonation of water by added base, and enabled total simulation times up to 60 ps. Even though the initial one-electron oxidation is much faster, these long simulation times were necessary to capture reactive trajectories without imposing artificial bias on the system.

**Fig. 2:**
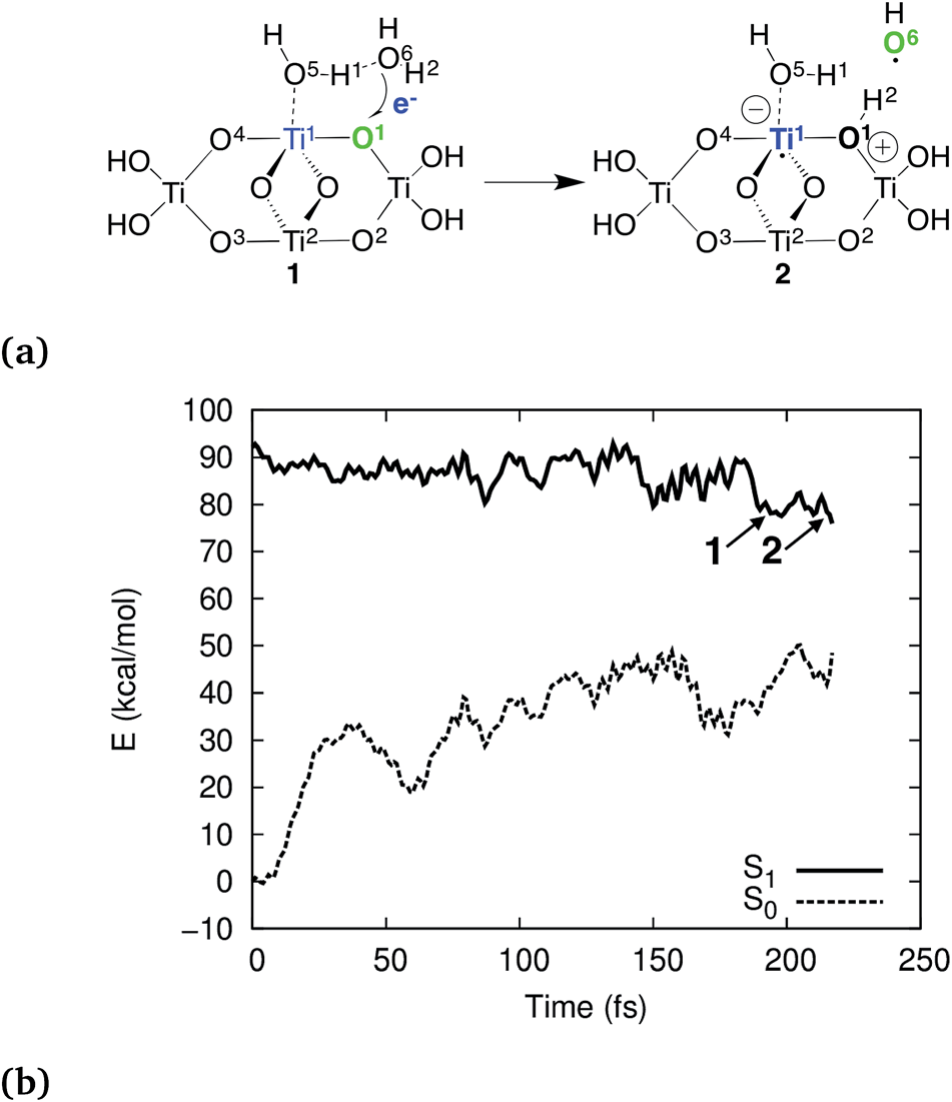
Schematic presentation of the observed EPT reaction (a) and the S_1_ and S_0_ PESs for the reactive trajectory for (TiO_2_)_4_(OH)_4_(H_2_O)_8_ (b). In (a), blue and green colors indicate the location of the electron and the hole, respectively.

According to our simulations, the reaction starts by electron–proton transfer (EPT) from physisorbed water to the photohole strongly localized on O_br_ as depicted in Fig. 2a. In (TiO_2_)_4_(OH)_4_(H_2_O)_8_, this reaction occurs on the S_1_ potential energy surface (PES), close to the S_1_–S_0_ conical intersection (CI), see Fig. 2b. The reactive intermediate **1** forms approximately 200 fs after the trajectory was initiated and undergoes hydrogen-transfer reaction within 20 fs to form intermediate **2**.

**Fig. 3:**
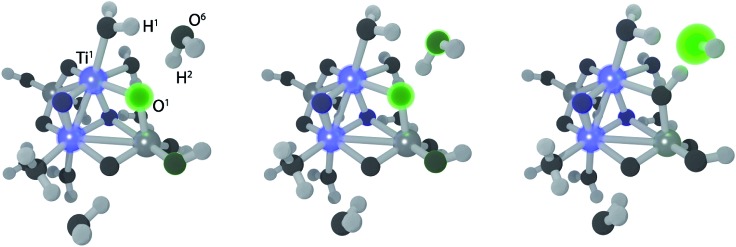
Snapshots from a NAMD trajectory at 200 fs (left), 213 fs (middle) and 218 fs (right) showing EPT for (TiO_2_)_4_(OH)_4_(H_2_O)_8_. Blue and green colors indicate negative and positive computed excitonic (electron–hole pair) charges, respectively.

The electron transfer reaction is displayed in Fig. 3 for (TiO_2_)_4_(OH)_4_(H_2_O)_8_. At 200 fs, the photohole (green) is localized strongly on bridging oxygen O^1^, forming the intermediate **1**. The hole is transferred to physisorbed water at 213 fs and intermediate **2** forms at 219 fs *via* concerted proton transfer without substantial nuclear reorientation. Intermediate **2** subsequently decays rapidly to S_0_ through a CI and forms a stable ground state intermediate followed by dissociation of the hydroxyl radical (see ESI†).

The structure of the intermediate observed in the NAMD simulations is in close agreement with STM measurements.^22^ Moreover, our mechanism yields mobile OH radicals, in accordance with several recent experiments.^21,23,24^ The charge-transfer (CT) reactivity seen in our simulations is indirectly supported by a TS study of Ti(OH)_4_^28^ and NAMD simulations of the oxidation of chemisorbed methanol on a TiO_2_ surface.^38^

**Fig. 4:**
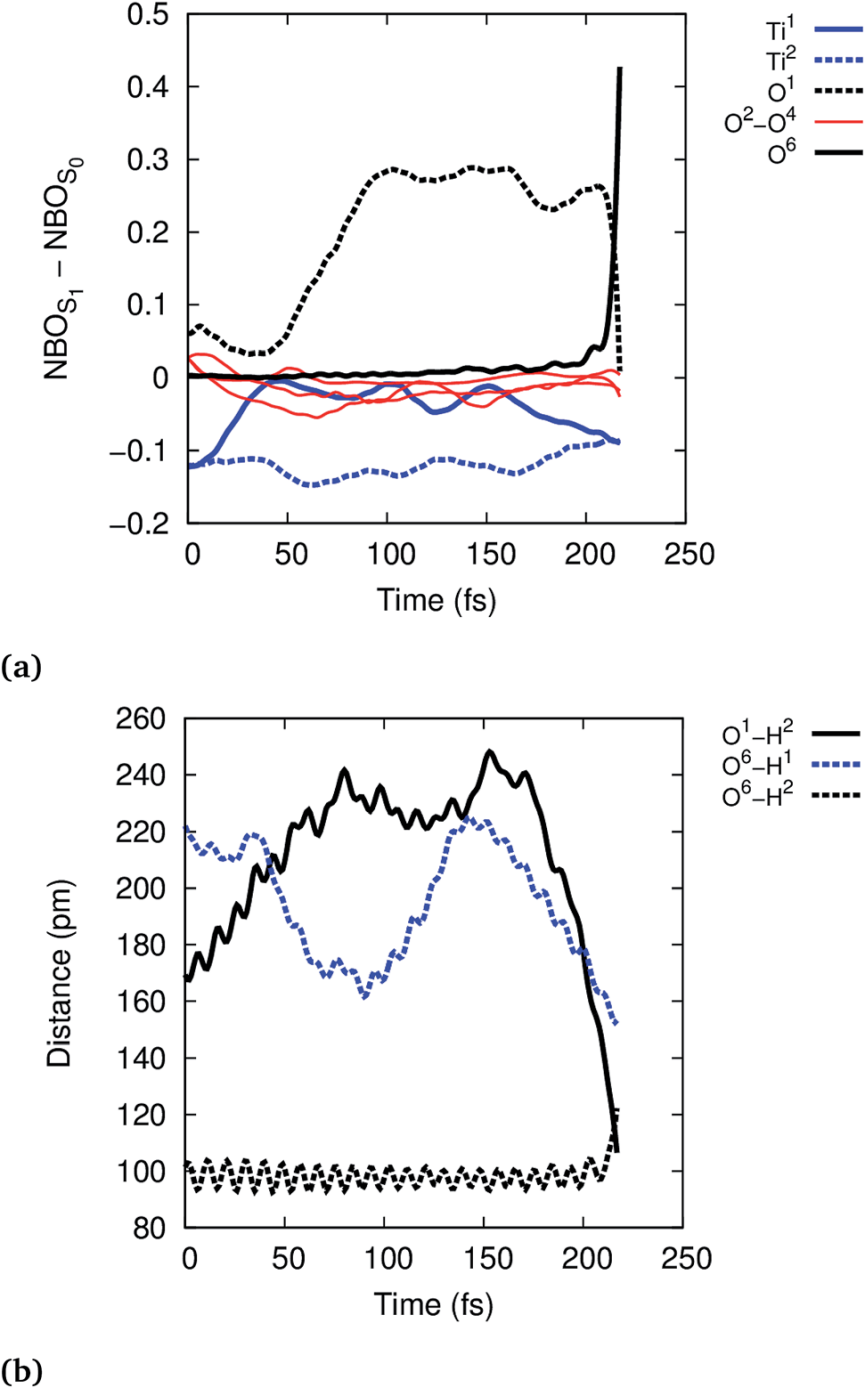
Time-evolution of the exciton according to NBO population analysis (a) and of the selected O–H distances (b) in the reactive trajectory for (TiO_2_)_4_(OH)_4_(H_2_O)_8_ nanoparticle.

To further analyze the exciton dynamics and the resulting EPT, we consider the difference in atomic natural bonding orbital^39^ (NBO) charges between the S_1_ and the S_0_ states, see Fig. 4a. Positive values of the population difference indicate hole charge, *i.e.*, loss of electron density on atoms relative to the ground state, and negative values indicate electron charge, *i.e.*, gain of electron density relative to the ground state. In the Franck–Condon geometry, the hole is shared between all bridging oxygens, O^1^–O^4^, and the electron is distributed equally to Ti^1^ and Ti^2^. During the first 100 fs, the hole localizes strongly on the bridging oxygen O^1^ until EPT from the physisorbed water H_2_O^6^ occurs at 213 fs. The other bridging oxygens, O^2^–O^4^, gain electron density and thus act as electron traps. This allows them to hydrogen bond with liquid water more efficiently, but does not lead to any reactivity. A consequence of the hole localization is the subsequent localization of the electron on Ti^1^ adjacent to the reactive O^1^,
7

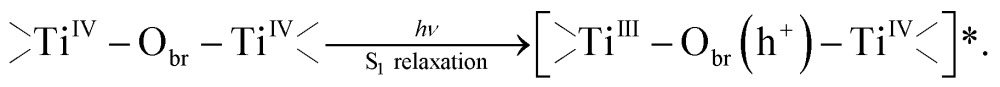




The localization of the two opposite charges provides coulombic stabilization of the exciton and drives the reaction

8 [Ti^III^–O_br_(h^+^)–Ti^IV^
]* + H_2_O(l) → [Ti^III^–(O_br_H)^+^–Ti^IV^
 + ·OH]*

to form Ti^III^–(O_br_H)^+^–Ti^IV^  species, which are stable on a picosecond timescale in *ab initio* molecular dynamics simulations.^40^ While less confinement may increase the exciton size, the energy gain from localization also increases in larger particles. Larger rutile hydrated and hydroxylated (TiO_2_)_23_ nanoparticles exhibit exciton localization after self-trapping (*i.e.* at the S_1_ PES minimum) on a similar scale as the ones studied here,^41^ suggesting that the self-trapped exciton size may not depend strongly on the particle size.

Photohole localization is not the only driving force of the reaction, however, since EPT only occurs 100 fs after the photohole localizes: starting at ∼150 fs, the reactive physisorbed water hydrogen bonds more strongly with the Ti^1^ bound water (O^6^–H^1^ distance decreases from 220 pm to 170 pm), see Fig. 4b; concurrently, the electron starts to localize on Ti^1^, see Fig. 4a. The EPT follows these changes as seen in the O^1^–H^2^ and O^6^–H^2^ distances. This suggests that the localized electron on Ti^1^ also favors EPT by some electron transfer to the water bound to Ti^1^. The electron rich water then stabilizes the nascent H_2_O^+^ by solvating the hole, thus facilitating the reaction. This interpretation is also supported by the observation that the reaction is faster in the smaller (TiO_2_)_4_(OH)_4_(H_2_O)_4_ model: Ti^1^ is bound to only one water instead of two in (TiO_2_)_4_(OH)_4_(H_2_O)_8_, which facilitates electron localization and concomitant stabilization of the photohole by hydrogen bonding interaction. Intermediate **1** is formed approximately at 150 fs in the reactive trajectory for (TiO_2_)_4_(OH)_4_(H_2_O)_4_, similar to the reactivity observed for the larger (TiO_2_)_4_(OH)_4_(H_2_O)_8_ particle (Fig. S5 and S6†).

Why did previous simulations not show the present mechanism? These simulations were based on free charge carriers, *i.e.*, cationic and anionic species in the electronic ground state, which do not include electron–hole interaction. For (TiO_2_)_4_(OH)_4_(H_2_O)_8_, the reaction is exothermic by approximately 10 kcal mol^–1^ compared to the Franck–Condon geometry on the S_1_ PES (Fig. 2b). On the other hand, for a free hole the reaction is endothermic by approximately 7 kcal mol^–1^ (ESI†). This 17 kcal mol^–1^ difference is mainly due to coulombic stabilization of the exciton (“exciton binding energy”), of the H_2_O^+^ species, and of the protonated bridging oxygen O^1^ by the electron component of the exciton. This was confirmed by BOMD simulations for free holes which did not show any reactivity up to 135 ps of total simulation time. Explicit simulation of both, the electron and the hole and their interaction, *i.e.*, electronic excitation beyond the single-particle picture, is necessary to explain the reactivity. Furthermore,the oxidation occurs only after relaxation of the S_1_ state, and thus the reactivity cannot be rationalized from the Franck–Condon geometry.

## Conclusions

The first unconstrained NAMD simulations of water oxidation by small TiO_2_ nanoparticles show EPT from physisorbed liquid water to a strongly localized hole on O_br_. This mechanism is consistent with STM experiments,^22^ and generates mobile hydroxyl radicals in accordance with recent experiments based on three different fluorescence probe methods and total internal reflection fluorescence microscopy.^21,23,24^ The calculations reveal two key driving forces of the oxidation reaction: (i) localization of the exciton with close proximity of the electron and hole charges leads to a gain of coulombic stabilization. (ii) Simultaneously, hydrogen bonding stabilizes the emerging H_2_O^+^ species, which is deprotonated to free OH in the excited state.

These results provide a rationale for the low catalytic activity of TiO_2_ in water splitting: while exciton localization is necessary to drive the reaction, it can also promote recombination of the electron and hole charges, *i.e.*, non-radiative decay to the ground state. This is seen in the vast majority of our trajectories. Similarly, the effective stabilization of the photohole by hydrogen bonding requires a specific orientation of surface bound water, which has a large entropic penalty. While additional validation of the proposed mechanism is desirable, *e.g.*, by exploring the effects of the particle size and bulk solvation, the present results could inform future efforts to increase the water splitting activity of TiO_2_-based photocatalysts by targeted synthetic modification.
