# Supplementary material for: Mechanism of photocatalytic water oxidation on small TiO2 nanoparticles
Source: Chem Sci. 2016 Dec 7;8(3):2179–83. doi: 10.1039/c6sc04378j (PMC5407260; doi:10.1039/c6sc04378j)
Supplement: Supplementary file 1 [file SC-008-C6SC04378J-s001.pdf]

Electronic Supplementary Material (ESI) for Chemical Science.
This journal is © The Royal Society of Chemistry 2016

Electronic Supplementary Information for

Mechanism of Photocatalytic Water Oxidation on Small TiO_2 Nanoparticles

Mikko Muuronen,*[a] Shane M. Parker,[a] Enrico Berardo,[b,c] Alexander Le,[a] Martijn A. Zwijnenburg,[c] and Filipp Furche*[a]

Contents

1 Computational Details **2**

1.1 Choice of the Functional 2

2 NAMD Simulations **4**

2.1 Photo-Oxidation Reactions 4

2.2 Formation of Ground State Hydroxyl Radical 5

2.3 Other Reactive Trajectories 8

2.4 Non-adiabatic $S_1 \longrightarrow S_0$ Transitions 8

3 Free Carrier Simulations **10**

[a] *Department of Chemistry, University of California, Irvine, 1102 Natural Sciences II, Irvine, CA 92697-2025; Fax: +1 949 824 8571; Tel: +1 949 824-5051; E-mail: mmuurone@uci.edu, filipp.furche@uci.edu*
[b] *Department of Chemistry, Imperial College London, South Kensington, London, SW7 2AZ, U.K.*
[c] *Department of Chemistry, University College London, 20 Gordon Street, London WC1H 0AJ, U.K.*

1 Computational Details

Unless otherwise noted, all non-adiabatic and Born-Oppenheimer molecular dynamics (NAMD and BOMD) simulations used the PBE0[1] hybrid functional with D3[2] dispersion corrections, along with polarized double-ζ valence def2-SVP[3] basis sets and DFT integration grid 3. For a comprehensive discussion of the choice of the functional, see Section 1.1. The default settings of TURBOMOLE were used except the convergence treshold for the electron density was increased (denconv 1d-7). To speed up the simulations, we used the multipole-accelerated resolution-of-identity approximation (MARIJ) for Coulomb contributions with corresponding auxiliary basis set for ground states,[4,5] and nonorthonormal Krylov space methods for excited states.[6]

We also performed BOMD simulations for systems with only the hole present as a comparison for our NAMD simulations. In these, an electron was removed from the system and simulations were performed in the electronic doublet state. The initial conditions for these simulations were identical to those for NAMD simulations while trajectories were propagated for up to 4 ps.

The structures were obtained by adding water molecules to the previously published global minima structures for small TiO_2 nanoparticles.[7] The particles represent ideal surfaces in which all titanium atoms are in formal oxidation state +IV. The hydrated systems were then optimized using the meta-GGA functional TPSS-D3[2,8] in water, in which solvation effects were accounted for using COSMO[9,10] solvation model ($\varepsilon = 80.1$). Next, the optimized systems were equilibrated at the same level using BOMD for 3 ps at 350 K. In these simulations, the temperature was controlled using a Nose-Hoover thermostat. We used the TPSS functional here because it has been shown to be accurate for ground state energies throughout the periodic table and,[11] by virtue of requiring no Hartree-Fock exchange (HFE), TPSS calculation with MARIJ approximation is approximately an order of magnitude faster than hybrid density functional calculation.

1.1 Choice of the Functional

Inaccuracies of the TDDFT potential energy surfaces (PES) can strongly affect the NAMD simulations reported here.[12,13] The performance of density functional approximations (DFAs) for the S_1 PES of TiO_2 nanoparticles depends considerably on the amount of HFE employed:[7,14] Nonhybrid DFAs (0% of HFE) spuriously overstabilize charge transfer (CT) excitations.[12] This effect is mitigated with global and range-separated hybrid density functionals, but not entirely removed.

To assess the sensitivity of our simulations to the computed PES, we performed excited state calculations for ten random conformers using two density functionals—PBE0-D3 (25% HFE) and BHLYP-D3 (50% HFE)—and compared the resulting energies and characters of the excited states. The ten conformers were obtained from ground state BOMD simulations of a neutral $(TiO_2)_4(OH)_4(H_2O)_8$ nanoparticle at 350 K. The consistent character of the excitations ($n \longrightarrow d$ type, no CT character, see Figure S1) with differing fractions of HFE indicates that PBE0 calculations are not contaminated with spurious CT states.

While the two functionals provide consistent character of the S_1 PES, the increased amount of HFE affects the relative energies significantly, see Figure S2 (left). The energy differences of PBE0-D3 and

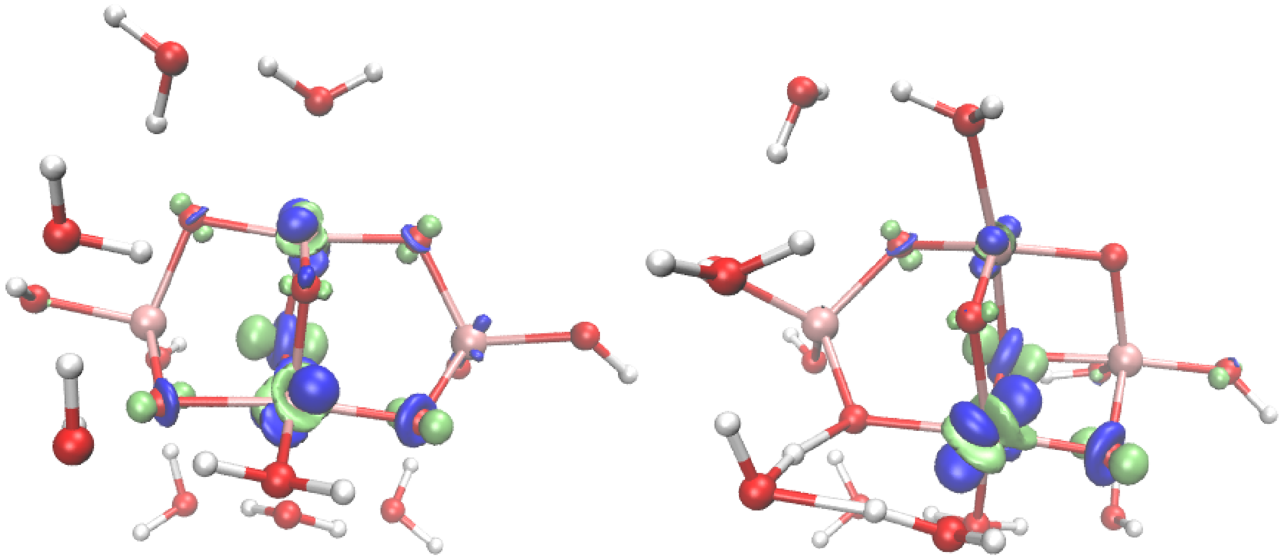

Fig. S1 Relaxed electron density difference between the S_1 and S_0 states to visualize the hole (green) and the electron (blue) for random conformers 1 (left) and 7 (right) at PBE0-D3/def2-SVP level (countour values of ± 0.015). BHLYP-D3 density differences are very similar.

BHLYP-D3 in the S_1 and S_0 PESs are already visible on the S_0 PES: The energies relative to conformer 1 are systematically higher for BHLYP-D3 than PBE0-D3. Figure S2 compares the BHLYP-D3 and PBE0-D3 S_0 energies to those obtained from resolution-of-identity random phase approximation (RI-RPA) [15–18] calculations, which we consider a benchmark since RI-RPA has been shown to be accurate for transition metal compounds . [19,20] For RPA, we used PBE [21] orbitals, def2-TZVP basis sets, [3,5,22] and core orbitals were kept frozen for the computation of correlation energy.

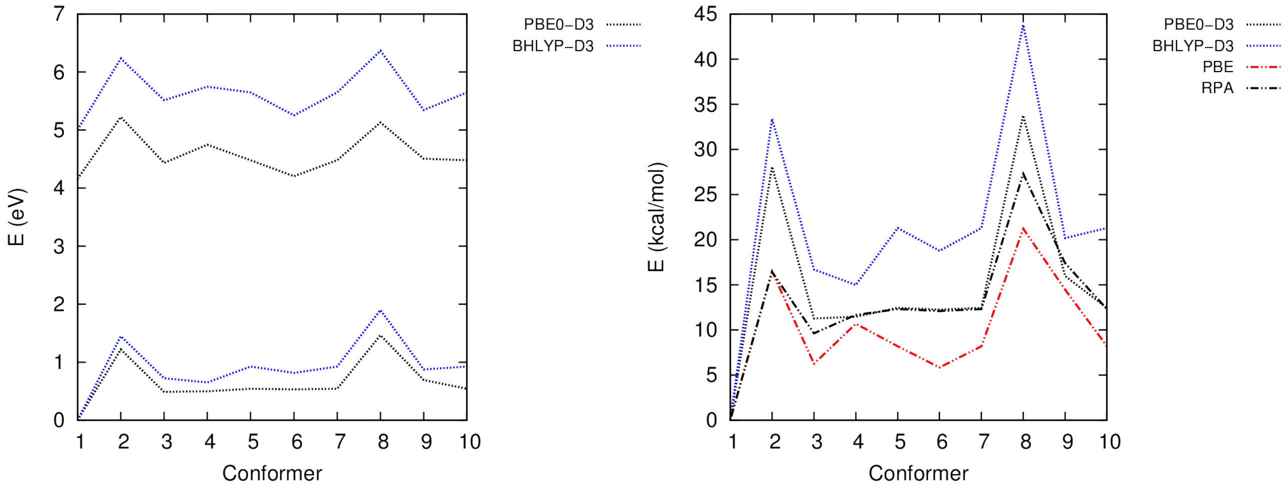

Fig. S2 The relative energies S_1 and S_0 electronic states for 10 random conformers of $(TiO_2)_4(OH)_4(H_2O)_8$ nanoparticle for PBE0-D3 and BHLYP-D3 functionals (left); and the same S_0 relative energies also using PBE and RPA (right).

Overall, PBE0-D3 performs well for the S_0 PES with two exceptions: The relative energy of conformers 2 and 8 are overestimated by approximately 10 and 6 kcal/mol respectively. For those same conformers, BHLYP-D3 produces still higher energies (up to 20 kcal/mol greater than RI-RPA) while the nonhybrid PBE functional underestimates the energies relative to RI-RPA by up to 5 kcal/mol.

To balance avoiding contamination by spurious CT states on the one hand and maintaining an accurate description of the ground state PES on the other, we chose PBE0 for our final analyses. Furthermore, possibility for contamination with CT states during PBE0 trajectories was examined by computing single-point energies with BHLYP-D3 at several structures along the PBE0 trajectory and comparing the effect of HFE on the structure of the exciton and the PES.

2 NAMD Simulations

NAMD simulations were performed for small hydrated $(TiO_2)_4(OH)_4 \cdot (H_2O)_n$ nanoparticle with $n = 2, 4, 8$ and 10 (Figure S3). A total of 115 independent trajectories were run, each starting from a random initial structure as described in Section 1 and running for up to 1 ps. Due to nonradiative decay, however, the average simulation time was $\sim$ 0.5 ps. Thus, the aggregate exciton simulation time was $\sim$ 60 ps.

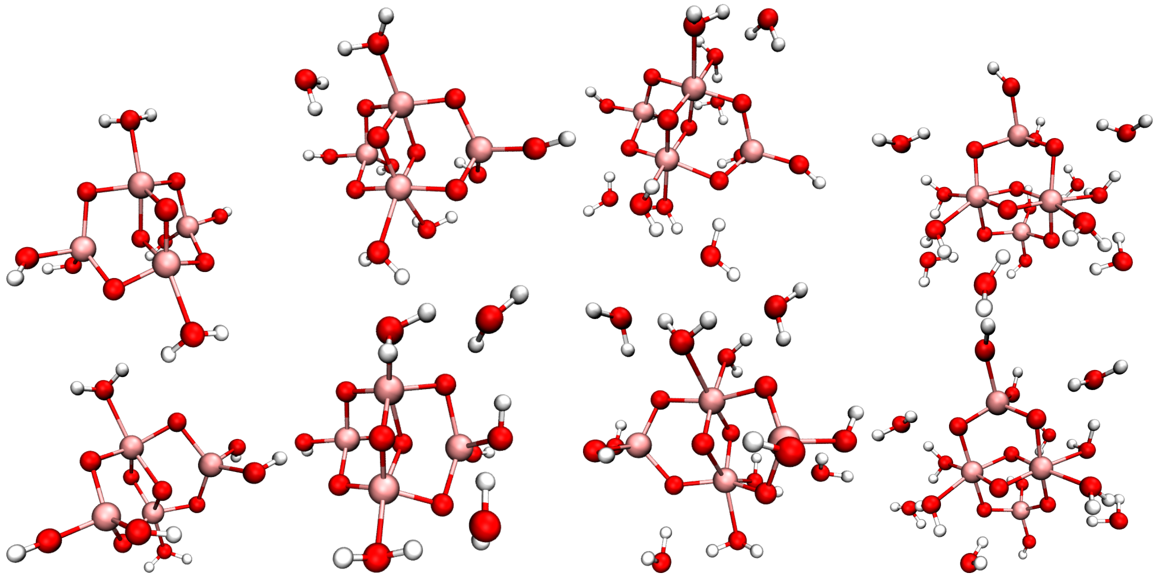

Fig. S3 Studied $(TiO_2)_4(OH)_4$ nanoparticles with 2, 4, 8 and 10 additional water molecules from left to right, respectively.

2.1 Photo-Oxidation Reactions

Among 115 trajectories, two exhibited clear photo-oxidation reactivity. The reactive trajectory for $(TiO_2)_4(OH)_4(H_2O)_8$ is analyzed comprehensively in the main text, and the corresponding data for $(TiO_2)_4(OH)_4(H_2O)_4$ is presented in Figures S4-S6. Similarly to observed reaction for $(TiO_2)_4(OH)_4(H_2O)_8$, the reaction takes place close to the S_1-S_0 conical intersection (CI). The reaction requires strong localization of the hole on O_{br}, and is triggered by localization of the electron on the adjacent Ti^1. $\cdot$OH is formed in the S_1 state in both trajectories.

Both reactive trajectories were also analyzed using a series of single point calculations with the BHLYP-D3 functional to exclude spurious charge transfer being the origin of the observed reactivity, see Figures S7-S8. While in the case of BHLYP-D3 we observe a slightly smaller change in S_1 energy along the trajectory, the time-evolution of the exciton localization, as analyzed in terms of the difference between S_0 and S_1 NBO charges, is very similar for the PBE0-D3 and BHLYP-D3 calculations,

suggesting that the qualitative result obtained is not sensitive to the choice of functional.

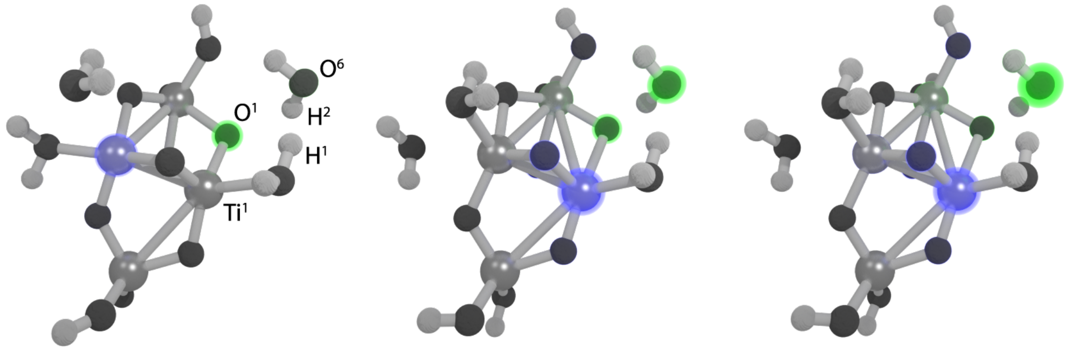

Fig. S4 The observed EPT transfer mechanism for $(TiO_2)_4(OH)_4(H_2O)_4$ nanoparticle: 120 fs (left), 160 fs (middle) and 164 fs (right). Blue and green colors indicate negative and positive computed excitonic charges due to electron and hole localization, respectively.

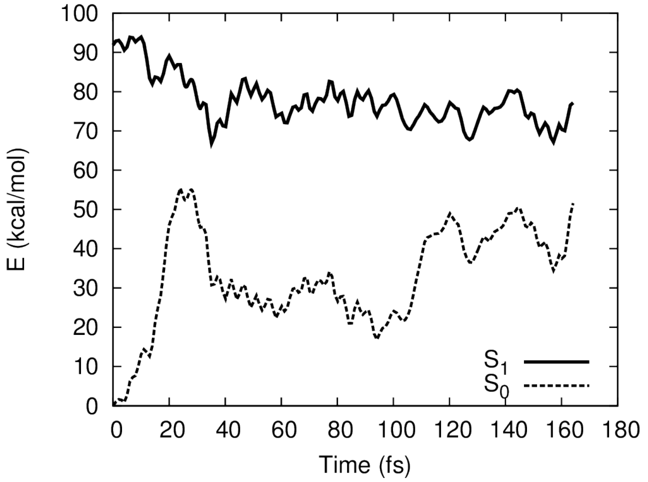

Fig. S5 The S_1 and S_0 PESs for the reactive trajectory for $(TiO_2)_4(OH)_4(H_2O)_4$.

2.2 Formation of Ground State Hydroxyl Radical

We avoid convergence and reference stability problems close to CIs by forcing a hop to the ground state whenever the optical gap falls below 0.5 eV.[23] In both reactive trajectories, a CI is approached shortly after the photohole transfers to water and the forced hop leads to a reversal of the photohole transfer.

We probed the sensitivity of the results to the location of the CI and the effect of forced hops by restarting 20 trajectories with randomized velocities along the excited state surface from the locations of the forced hops but now used BHLYP-D3 forces and energies, and forced a hop whenever the optical gap falls below 0.1 eV. As seen in Figure S2, the excited state surfaces computed with BHLYP-D3 are shifted up in energy relative to the surfaces computed with PBE0-D3, but are otherwise similar. No exciton recombination was seen in any of these restarted trajectories. Figures S9 and S10 show a restarted trajectory in which ·OH is formed 9 fs after the (re)start and the spin symmetry is broken after 80 fs.

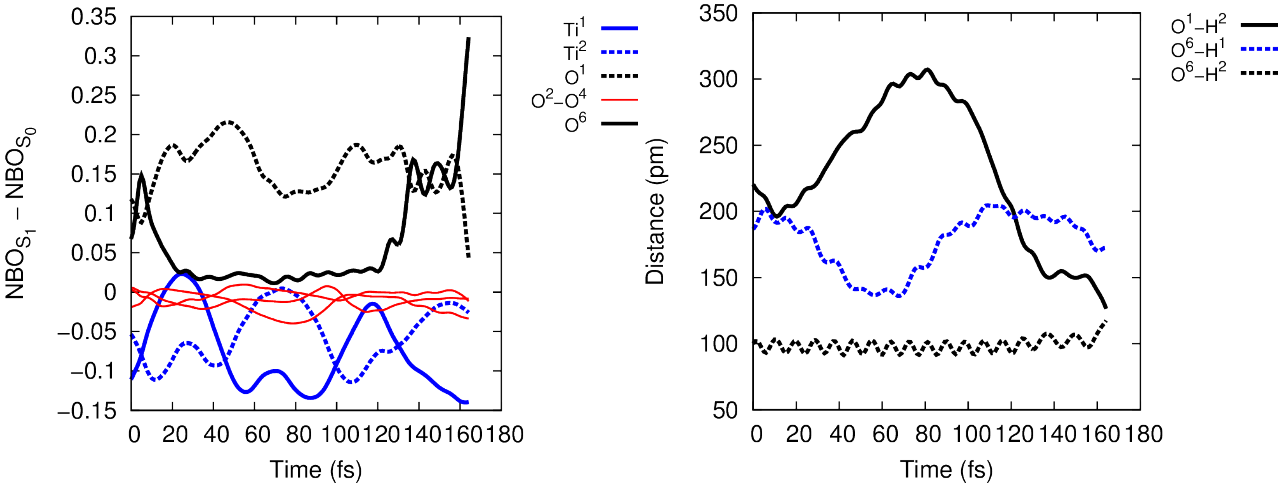

Fig. S6 Time-evolution of the exciton according to NBO population analysis (left) and of the selected O-H distances (right) in the reactive trajectory for $(TiO_2)_4(OH)_4(H_2O)_4$ nanoparticle.

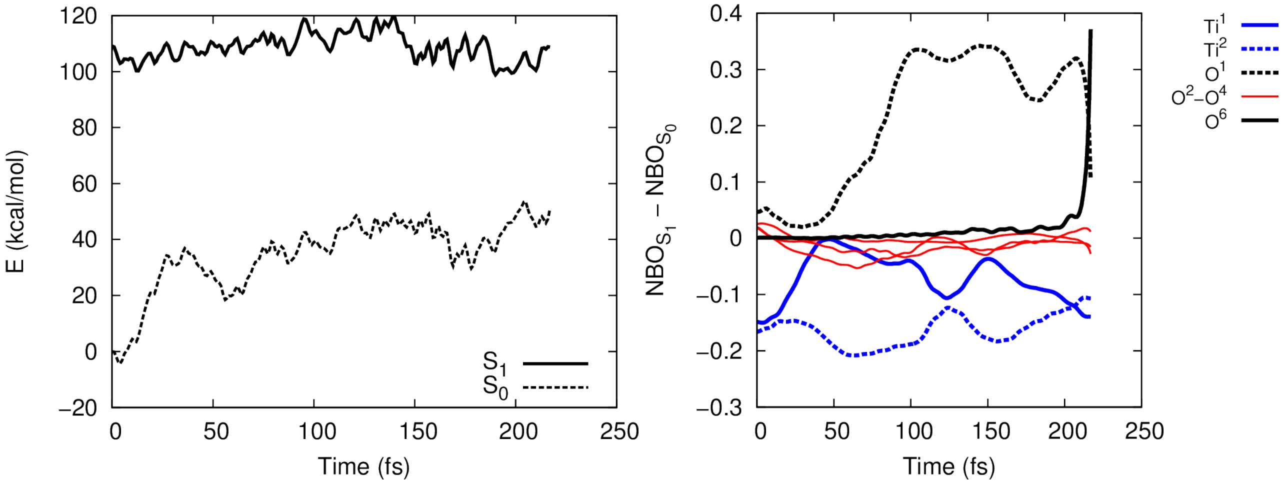

Fig. S7 The PES (left) and the time-evolution of the exciton according to NBO population analysis (right) in the reactive trajectory for $(TiO_2)_4(OH)_4(H_2O)_8$ nanoparticle obtained using BHLYP-D3 functional for a series of single point calculations on a PBE0-D3 trajectory.

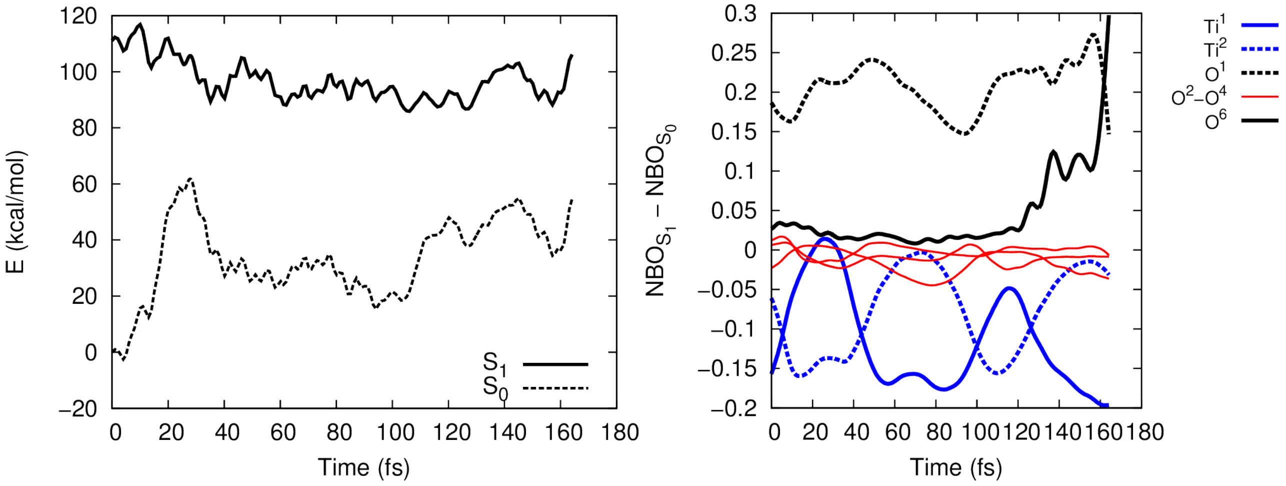

Fig. S8 The PES (left) and the time-evolution of the exciton according to NBO population analysis (right) in the reactive trajectory for $(TiO_2)_4(OH)_4(H_2O)_4$ nanoparticle obtained using BHLYP-D3 functional for a series of single point calculations on a PBE0-D3 trajectory.

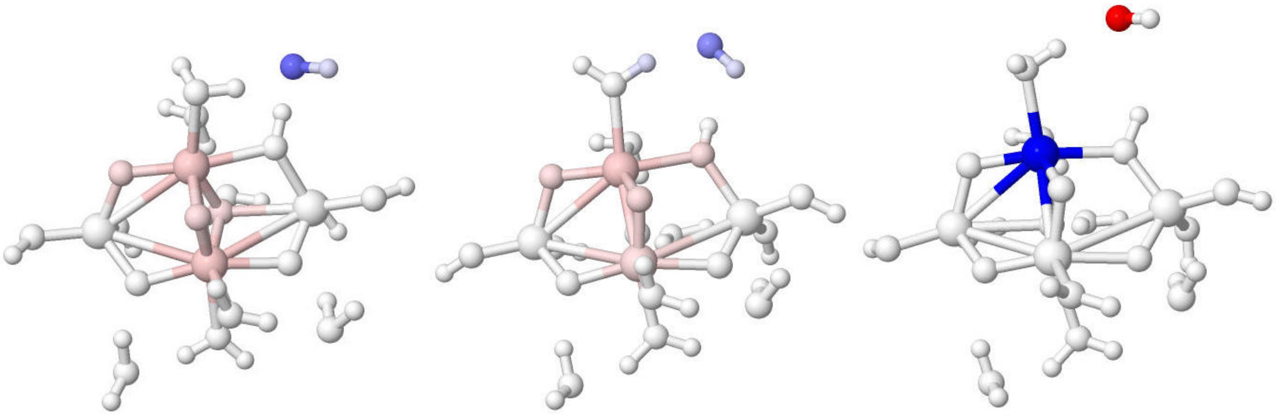

Fig. S9 The decay mechanism of the radical for the $(TiO_2)_4(OH)_4(H_2O)_8$ nanoparticle: 9 fs (left), 50 fs (middle) and 80 fs (right). Left and middle: The electron and the hole of the exciton are visualized using red and blue colors according to NBO charge difference between S_1 and S_0, respectively. Right, the atoms are colored by the NBO spin density.

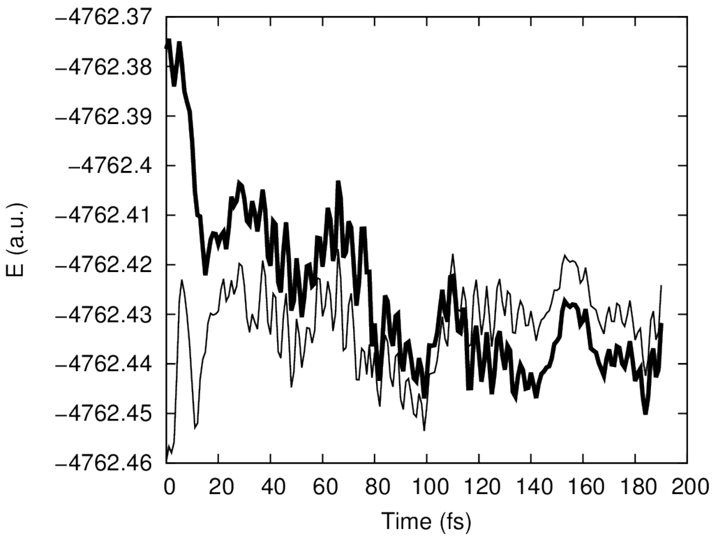

Fig. S10 The S_1 and S_0 PESs for the radical decay mechanism for $(TiO_2)_4(OH)_4(H_2O)_8$ nanoparticle. The propagated state is presented as bold.

2.3 Other Reactive Trajectories

We observed two mechanisms which do not involve photo-oxidation or may be be computational artifacts. Thus, these are are only discussed here.

The first mechanism not considered further comprises thermally hot reactions in the S_0 PES after the surface hop. For example trajectory, see Figures S11-S12. In this trajectory, the hole is delocalized over O_{br}, the terminal hydroxyl group, and terminal titanium at 250 fs (Figure S11 (left)). This leads to a non-adiabatic $S_1 \longrightarrow S_0$ transition (Figure S12), generating charged Ti^+ and $Ti-O^-$ species in the ground state. Approximately 30 fs later, the $Ti-O^-$ deprotonates surface bound water to generate a defected neutral surface via hot acid-base reaction. Thus, this is not an oxidation reaction. Furthermore, these reactions requires strongly distorted structures that are only possible due to the small particle size employed in our simulations where atoms are less constrained in comparison to larger nanoparticles or bulk systems.

In the second mechanism, the ·OH is formed as mobile species from the terminal surface bound OH immediately after the initial excitation. This mechanism originated from initial CT excitation and was not reproduced with the BHLYP functional.

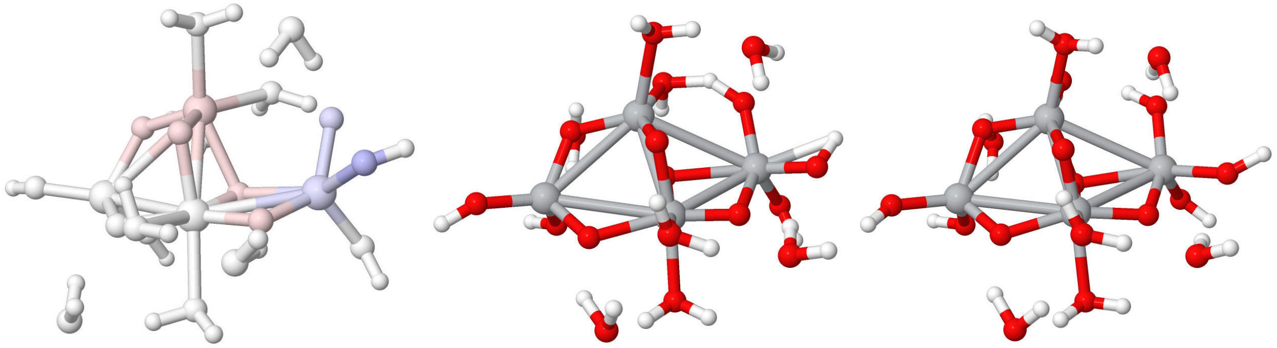

Fig. S11 Snapshots for $(TiO_2)_4(OH)_4(H_2O)_8$ reactive trajectory taking place in S_0 PES: 250 fs (left), 282 fs (middle) and 301 fs (right). For 250fs, the exciton is visualized with red and blue for electron and hole, respectively, according to the NBO charge difference between S_1 and S_0.

2.4 Non-adiabatic $S_1 \longrightarrow S_0$ Transitions

Beyond the reactive trajectories, the unreactive trajectories help establish a fundamental understanding of the atomistic mechanism of the nonradiative $S_1 \longrightarrow S_0$ transitions that lead to exciton annihilation, and thus limit the efficiency of titania based photocatalysts.[24] These are observed mainly close to conical intersections where the hole is localized strongly on O_{br} and the electron localizes to the adjacent Ti. For a representative trajectory for $(TiO_2)_4(OH)_4$ without additional water molecules, see Figure S13. The Coulombic stabilization allows stretching of the Ti-O bond and leads to S_1-S_0 conical intersection, and to the decay of the S_1 excited state. Thus, the non-adiabatic $S_1 \longrightarrow S_0$ transitions originate from identical exciton structure to the photo-oxidations.

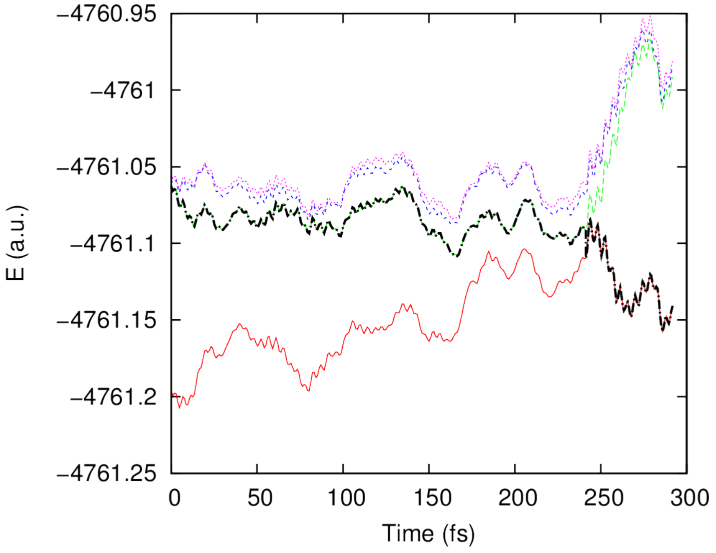

Fig. S12 The PESs of trajectory presented in Figure S11 for $(TiO_2)_4(OH)_4(H_2O)_8$. The propagated state is presented with bolded line ($S_1 \longrightarrow S_0$ at 250 fs).

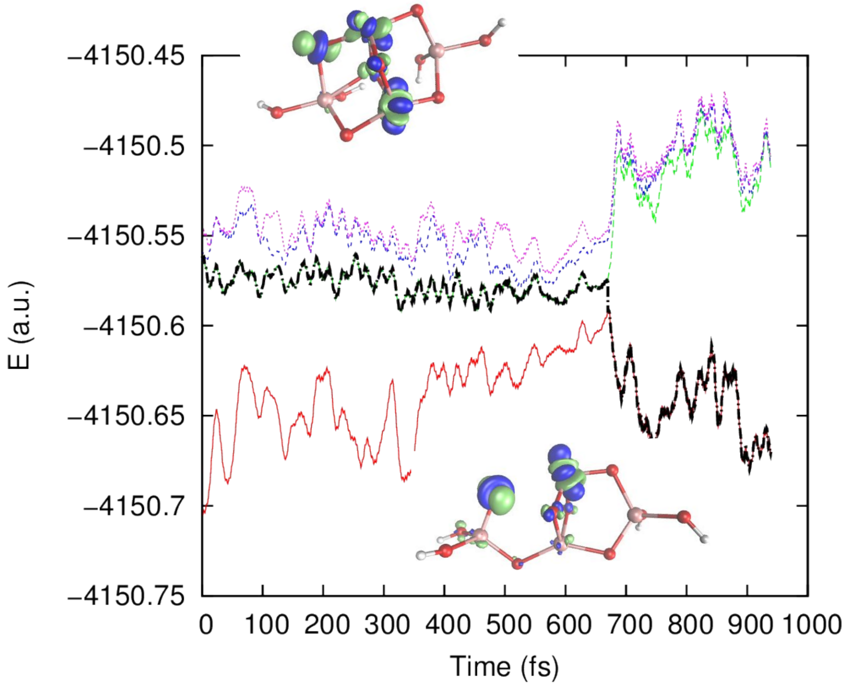

Fig. S13 A representative trajectory of the exciton decay for $(TiO_2)_4(OH)_4$. The snapshots present electron density difference between S_1 and S_0: The hole is visualized with green, and the electron with blue. See Figure S1 for details.

3 Free Carrier Simulations

To simulate a free carrier, we performed simulations $(TiO_2)_4(OH)_4(H_2O)_8$ and $(TiO_2)_4(OH)_4(H_2O)_{10}$ nanoparticles in which only the hole is present, i.e., we removed an electron from the system. The results are based on 35 independent trajectories, which were run for 4 ps each. Thus, the total simulation time was 135 ps. To investigate solvation effects, half of the simulations were performed in gas phase and half with COSMO. We did not observe this to influence the reactivity nor dynamics significantly, i.e., the hole localization is qualitatively similar during the simulations, as analyzed from NBO spin densities. Representative snapshots showing the localization of the hole are displayed in Figure S14.

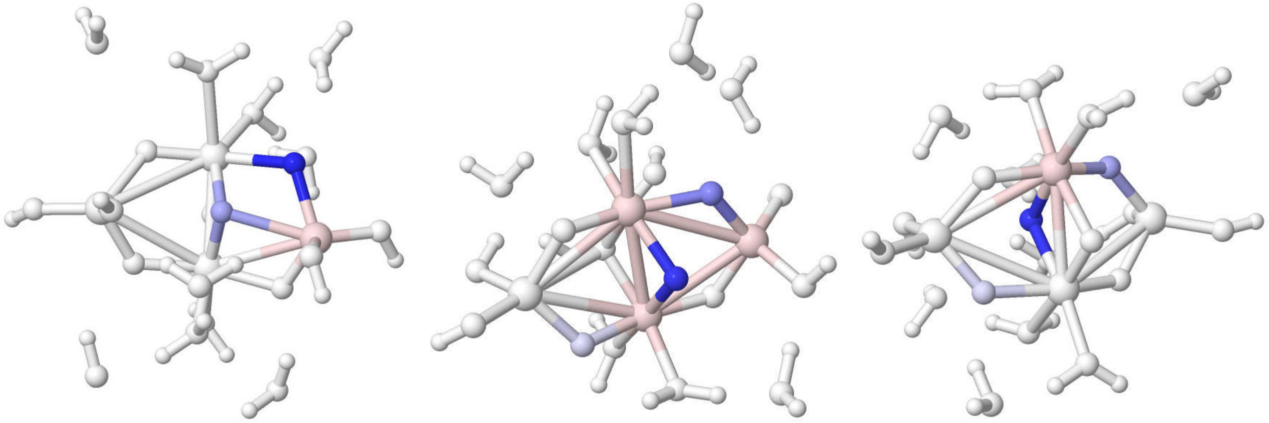

Fig. S14 Snapshots of BOMD trajectories for $(TiO_2)_4(OH)_4(H_2O)_{10}$ free carrier. Atoms are colored according to NBO spin densities, and the hole is presented as blue.

We also studied the potential energy for the reactive trajectory using $(TiO_2)_4(OH)_4(H_2O)_8{}^+$. To this end, we calculated the energy of the corresponding nuclear geometries as charged species. The PESs for exciton (S_1) and free carrier (FC) are compared in Figure S15. While reaction is exothermic by $\sim$ 10 kcal/mol when the neutral species is electronically excited, it is endothermic by $\sim$ 7 kcal/mol when only the hole is present.

References

1 J. P. Perdew, M. Ernzerhof and K. Burke, *J. Chem. Phys.*, 1996, **105**, 9982.

2 S. Grimme, J. Antony, S. Ehrlich and H. Krieg, *J. Chem. Phys.*, 2010, **132**, 154104.

3 F. Weigend and R. Ahlrichs, *Phys. Chem. Chem. Phys.*, 2005, **7**, 3297–3305.

4 M. Sierka, A. Hogekamp and R. Ahlrichs, *J. Chem. Phys.*, 2003, **118**, 9136.

5 F. Weigend, *Phys. Chem. Chem. Phys.*, 2006, **8**, 1057–1065.

6 F. Furche, B. T. Krull, B. D. Nguyen and J. Kwon, *J. Chem. Phys.*, 2016, **144**, 174105.

7 E. Berardo, H.-S. Hu, H. J. J. van Dam, S. A. Shevlin, S. M. Woodley, K. Kowalski and M. A. Zwijnenburg, *J. Chem. Theory Comput.*, 2014, **10**, 5538–5548.

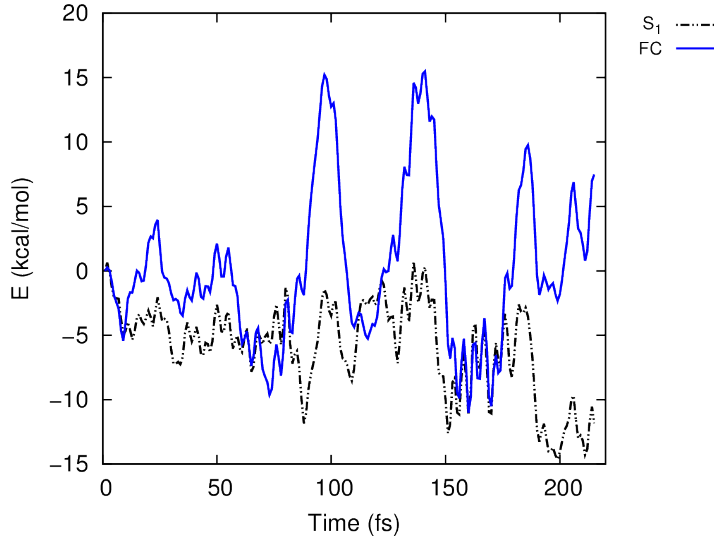

Fig. S15 The PES of the reactive trajectory for $(TiO_2)_4(OH)_4(H_2O)_8$, described as exciton (S_1) and free carrier (FC).

8 J. Tao, J. Perdew, V. Staroverov and G. Scuseria, *Phys. Rev. Lett.*, 2003, **91**, 146401.

9 A. Klamt and G. Schüürmann, *J. Chem. Soc., Perkin Trans. 2*, 1993, 799–805.

10 A. Schäfer, A. Klamt, D. Sattel, J. C. W. Lohrenz and F. Eckert, *Phys. Chem. Chem. Phys.*, **2**, 2187–2193.

11 L. Goerigk and S. Grimme, *Phys. Chem. Chem. Phys.*, 2011, **13**, 6670–6688.

12 A. Dreuw and M. Head-Gordon, *J. Am. Chem. Soc.*, 2004, **126**, 4007–4016.

13 B. G. Levine, C. Ko, J. Quenneville and T. J. Martínez, *Mol. Phys.*, 2006, **104**, 1039–1051.

14 E. Berardo, H.-S. Hu, S. A. Shevlin, S. M. Woodley, K. Kowalski and M. A. Zwijnenburg, *J. Chem. Theory Comput.*, 2014, **10**, 1189–1199.

15 F. Furche, *J. Chem. Phys.*, 2008, **129**, 114105.

16 H. Eshuis, J. Yarkony and F. Furche, *J. Chem. Phys.*, 2010, **132**, 234114.

17 H. Eshuis, J. E. Bates and F. Furche, *Theor. Chem. Acc.*, 2012, **131**, 1084.

18 H. Eshuis and F. Furche, *J. Phys. Chem. Lett.*, 2011, **2**, 983–989.

19 J. E. Bates and F. Furche, *J. Chem. Phys.*, 2013, **139**, 171103.

20 M. P. Johansson, I. Warnke, A. Le and F. Furche, *J. Phys. Chem. C*, 2014, **118**, 29370–29377.

21 J. P. Perdew, K. Burke and M. Ernzerhof, *Phys. Rev. Lett.*, 1996, **77**, 3865–3868.

22 F. Weigend, M. Häser, H. Patzelt and R. Ahlrichs, *Chem. Phys. Lett.*, 1998, **294**, 143–152.

23 E. Tapavicza, G. D. Bellchambers, J. C. Vincent and F. Furche, *Phys. Chem. Chem. Phys.*, 2013, **15**, 18336–18348.

24 B. Ohtani, *Catalysts*, 2013, **3**, 942–953.
